# Serum Adiponectin Is Elevated in Critically Ill Patients with Liver Disease and Associated with Decreased Overall Survival

**DOI:** 10.3390/biomedicines12102173

**Published:** 2024-09-25

**Authors:** Maike R. Pollmanns, Qendrim Pajaziti, Philipp Hohlstein, Jule K. Adams, Samira Abu Jhaisha, Elena Kabak, Karim Hamesch, Sophie H. A. Nusser, Ralf Weiskirchen, Theresa H. Wirtz, Alexander Koch

**Affiliations:** 1 Department for Gastroenterology, Metabolic Disorders and Intensive Care Medicine, RWTH-University Hospital Aachen, Pauwelsstraße 30, 52074 Aachen, Germany; mpollmanns@ukaachen.de (M.R.P.); qendrim.pajaziti@rwth-aachen.de (Q.P.); phohlstein@ukaachen.de (P.H.); jadams@ukaachen.de (J.K.A.); sabujhaisha@ukaachen.de (S.A.J.); ekabak@ukaachen.de (E.K.); khamesch@ukaachen.de (K.H.); snusser@ukaachen.de (S.H.A.N.); thwirtz@ukaachen.de (T.H.W.); 2 Institute of Molecular Pathobiochemistry, Experimental Gene Therapy and Clinical Chemistry (IFMPEGKC), RWTH University Hospital Aachen, Pauwelsstraße 30, 52074 Aachen, Germany; rweiskirchen@ukaachen.de

**Keywords:** adiponectin, adipokine, intensive care unit (ICU), critical illness, acute liver failure (ALF), decompensated advanced chronic liver disease (dACLD), acute-on-chronic liver failure (ACLF), prognosis, liver transplantation

## Abstract

Background: Adiponectin, an adipokine with anti-inflammatory properties, has been implicated in various liver diseases. This study aimed to elucidate the prognostic value of serum adiponectin levels in critically ill patients with liver disease. Methods: This observational study included 161 critically ill patients admitted to the medical ICU of RWTH Aachen University Hospital due to acute liver failure or decompensated advanced chronic liver disease. Serum adiponectin levels were measured at ICU admission and after 48 h. Clinical parameters and outcomes, including transplant-free survival, were analyzed. Results: Serum adiponectin concentrations were significantly elevated compared to healthy controls (*p* < 0.001). Levels were particularly high in patients with sepsis compared to those with gastrointestinal bleeding as the precipitating factor of acute decompensation (*p* = 0.045) and were higher in female patients (*p* = 0.023). Adiponectin concentrations correlated with the Model of End-Stage Liver Disease (MELD) score and Child–Pugh score. Multivariate analysis confirmed a significant correlation with total bilirubin (r = 0.292, *p* < 0.001) and serum sodium (r = −0.265, *p* = 0.028). Higher adiponectin concentrations were associated with a trend towards poorer 30- and 180-day survival. Cox regression analysis identified a significant association between increased adiponectin concentration and reduced transplant-free survival (*p* = 0.037), supported by a Kaplan–Meier analysis using a cutoff of 119 ng/mL (log-rank 5.145, *p* = 0.023). Conclusions: Elevated serum adiponectin concentrations are associated with liver dysfunction and poor outcomes in critically ill patients. Higher adiponectin levels at ICU admission may predict poorer transplant-free survival. Further research in larger, multicenter cohorts is warranted to validate these findings and explore the underlying mechanisms.

## 1. Introduction

Adipose tissue secretes various factors, known as adipokines, that modulate inflammatory responses [1,2,3,4]. Among these, adiponectin is a 30 kDa adipokine that plays a crucial role in metabolic processes, such as reducing the postprandial rise of plasma-free fatty acids and enhancing insulin-mediated suppression of hepatic glucose output. It strongly correlates with systemic insulin sensitivity and has anti-atherogenic properties [5]. Adiponectin binds to two specific receptors, AdipoR1 and AdipoR2, predominately expressed in the liver [6]. Hepatic stellate cells and Kupffer cells express both receptors and are key mediators in liver injury [7,8]. Through the activation of AMPK and PPARα pathways, AdipoR1 and AdipoR2 regulate fatty acid and glucose metabolism [9,10].

Adiponectin concentrations are lower in obese patients and inversely correlate with insulin resistance and blood pressure [5,11]. Decreased plasma adiponectin levels (hypoadiponectinemia) are more closely associated with insulin resistance and hyperinsulinemia than with adiposity and glucose intolerance [4]. Obesity, characterized by dysregulated adipokine secretion, is associated with chronic low-grade inflammation and an increased risk of liver disease, such as metabolic dysfunction associated with steatotic liver disease (MASLD) [1]. Serum adiponectin levels have been found to be inversely related to the presence of MASLD [12].

In patients with chronic liver disease, alterations in adipokine concentrations have been observed [13]. Adiponectin has protective effects against liver injury and fibrosis [14]. In chronic hepatitis C patients, high serum levels of adiponectin were associated with higher all-cause and liver-related mortality [15]. Adiponectin is negatively correlated with mediators of inflammation, including C-reactive protein (CRP) [16]. Hypoadiponectinemia promotes the activation of the inflammasome responsible for vascular endothelial dysfunction through a mechanism involving the formation of oxidative and nitrative stress [17].

Patients with acute deterioration of liver function often require treatment in the intensive care unit (ICU). Acute liver failure (ALF) occurs in individuals without prior chronic liver disease and is characterized by acute liver injury, an international normalized ratio (INR) above 1.5, and hepatic encephalopathy [18]. Chronic liver disease (CLD) encompasses conditions that cause progressive liver damage, often leading to cirrhosis (advanced CLD (ACLD)). CLD progresses from an asymptomatic phase (compensated ACLD, cACLD) to a symptomatic phase (decompensated ACLD, dACLD), resulting in hospitalization, impaired quality of life, and high mortality [19].

Acute-on-chronic liver failure (ACLF) involves acute deterioration of liver function accompanied by multiple organ failures and high short-term mortality in patients with chronic liver disease [20,21,22]. Currently, ACLF is diagnosed based on the Chronic Liver Failure Consortium ACLF-score (CLIF-C ACLFs) [20]. Higher ACLF grades indicate an increased number of organ failures and higher mortality risk, necessitating intensive therapeutic management [20]. Despite improvements in overall survival, ICU mortality remains high [23,24,25].

The regulation of adipokines in critically ill patients, especially those with ACLF, remains poorly understood. Previous studies presented conflicting findings regarding adiponectin levels in critically ill patients, with some showing levels similar to controls and others showing lower levels [13,26,27,28].

Given the role of adipokines in hepatic metabolism, liver regeneration, and systemic inflammation, it is promising to investigate serum adiponectin levels in critically ill patients with liver disease. This study aims to determine if there is a correlation between adiponectin levels and liver dysfunction and to explore the potential of adiponectin as a biomarker in these patients.

## 2. Materials and Methods

### 2.1. Study Design

The retrospective observational study was conducted at RWTH Aachen University Hospital, involving 161 patients admitted to the medical ICU between August 2015 and May 2021 due to advanced liver disease or acute liver failure. Informed consent was obtained from the patient, their spouse, or the appointed legal guardian upon ICU admission. Patients were eligible if they were above the age of 18 years with confirmed or suspected chronic liver disease or acute liver failure, as described previously [29]. Exclusion criteria included an anticipated ICU stay of less than 48 h, acute poisoning, or pregnancy. Acute liver failure (ALF) or acute-on-chronic liver failure (ACLF) was diagnosed based on the latest European Association for the Study of the Liver (EASL) guidelines [30,31]. A control group consisted of 20 healthy volunteers from the local blood bank, all of whom had regular blood counts and no clinical signs of acute infection or relevant chronic disease. The study was approved by the local ethics committee (EK 22-421, formerly EK 150/06) of the University Hospital RWTH Aachen, Germany, and adhered to the ethical principles outlined in the Declaration of Helsinki, 1964.

### 2.2. Adiponectin Measurements

Blood samples were taken upon ICU admission and again 48 h later. After a 10 min centrifugation at 4 °C, the serum samples were aliquoted and immediately stored at −80 °C for later analysis. Adiponectin concentrations in the serum were quantified using a commercially available enzyme-linked immunosorbent assay (ELISA) kit in accordance with the manufacturer’s instructions (Human Adiponectin/Arcp30 DuoSet ELISA, cat. No.: DY1065, R&D Systems, Bio-Techne Ltd., Abingdon, UK). All assays were conducted in a blinded manner, without access to the patient’s clinical or laboratory data.

### 2.3. Statistical Analysis

Statistical analysis and visualization were carried out using SPSS version 29 (SPSS, Chicago, IL, USA) and Python version 3.11. Given the skewed distribution of most variables, data are presented as median and range. Non-parametric tests were employed, with the Mann–Whitney U test or chi-squared test for comparing two groups and the Kruskal–Wallis test followed by a Dunn’s multiple comparison test or chi-squared test for more than two groups. The Wilcoxon signed-rank test was used for paired sample comparison. Correlations were evaluated using Spearman’s rank correlation test. After correlation analysis uni- and multivariable linear regression models were applied to assess the influence of covariates. Survival associations were analyzed using the Cox proportional hazards model, and Kaplan–Meier curves were used to display survival differences, with the log-rank test used to determine significance. A significance threshold of α = 0.05 was applied for all statistical calculations.

## 3. Results

### 3.1. Baseline Characteristics

We enrolled 161 critically ill patients with acute or chronic liver disease. The cohort included 20 patients with dACLD, 124 patients with ACLF, and 17 patients with ALF (Table 1). The median age of the patient cohorts showed no statistically significant differences. Patients with ACLF had a higher body mass index (BMI), with a median of 27.6, compared to a median BMI of 23.1 in the dACLD group and 25.7 in the ALF group (Table 1). Disease severity and organ failure, as indicated by the Acute Physiology and Chronic Health Evaluation II score (APACHE II) and the Sequential Organ Failure Assessment score (SOFA), were significantly higher in patients with ACLF (*p* < 0.001). Mechanical ventilation was required in 51.6% of patients with ACLF compared to 15% of patients with dACLD and 11.7% of patients with ALF (*p* < 0.001). Vasopressor use was significantly more frequent in patients with ACLF (80.6%) than in those with dACLD (5%) and ALF (23.5%) (*p* < 0.001). The model for end-stage liver disease (MELD) scores were higher in the ACLF and ALF groups, with medians of 28 and 30, respectively, compared to a median of 13 in the dACLD group (*p* < 0.001). The length of ICU stay was longer for patients with ACLF and ALF, with a median of 6 days for both groups, compared to a median of 3 days for the dACLD group (*p* < 0.001). Mortality rates were consequently higher in the ACLF group, both in the ICU (56.5% in ACLF, 0% in dACLD, and 23.5% in ALF, *p* < 0.001) and overall (1-year mortality of 66.7% in ACLF, 15.8% in dACLD, and 31.2% in ALF, *p* < 0.001, Table 1). Liver transplantation (LT) was more frequently performed in the ALF group (35.5%) compared to the dACLD (15%) and ACLF groups (10.5%) (*p* = 0.0199, Table 1).

### 3.2. Serum Levels of Adiponectin Are Elevated in Critically Ill Patients with Liver Dysfunction

**Figure 1:**
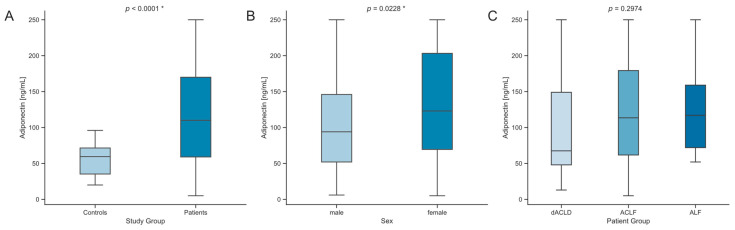
Comparison of serum adiponectin concentrations. (**A**) Serum adiponectin concentrations in controls compared to critically ill patients. Sample sizes: controls *n* = 20, patients *n* = 161. (**B**) Serum adiponectin concentrations in all study subjects in comparison between sexes. (**C**) Comparison of serum adiponectin concentrations between critically ill patients with dACLD, ACLF, and ALF. Sample sizes: dACLD *n* = 21, ACLF *n* = 124, ALF *n* = 17. Significance between groups was assessed using the Mann–Whitney U test (**A**,**B**) and Kruskal–Wallis test, followed by Dunn’s multiple comparison test (**B**). *p*-values less than 0.05 were considered statistically significant and were highlighted an asterisk (“*”). Abbreviations used include dACLD: decompensated advanced chronic liver disease; ACLF: acute-on-chronic liver failure; ALF: acute liver failure.

The median serum levels of adiponectin were significantly higher in patients with liver dysfunction compared to healthy controls (*p* < 0.0001, Figure 1A). Male patients had lower values of serum adiponectin than female patients (*p* = 0.0228, Figure 1B). Among these patients, those with dACLD had lower median serum adiponectin levels (67.5 ng/mL, Table 1) than patients with ACLF or ALF, although this difference was not statistically significant (Figure 1C). Serum adiponectin levels tended to be more elevated in higher grade ACLF determined by CLIF-C OF score without statistical significance (Figure S1). To investigate possible influences of preexisting diseases on serum adiponectin levels, comparisons were made across different groups, showing no significant differences (Table S1).

### 3.3. Impact of Decompensation Events in ACLD

**Figure 2:**
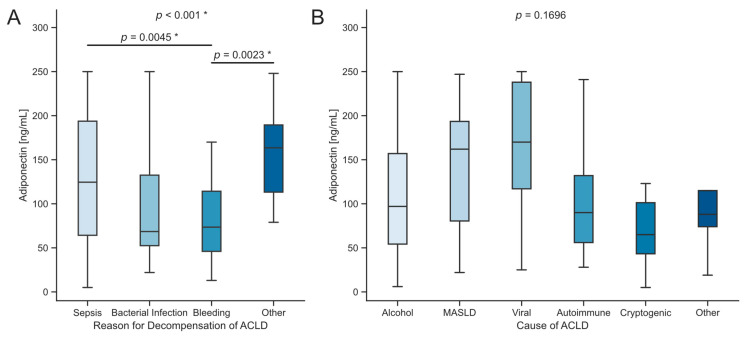
Serum adiponectin concentrations in advanced chronic liver disease. (**A**) Serum adiponectin concentrations compared to the reason for decompensation of ACLD. (**B**) Comparison of serum adiponectin concentrations among causes of ACLD. Sample sizes: dACLD *n* = 20, ACLF *n* = 124. Significance between groups was assessed using the Kruskal–Wallis test, followed by Dunn’s multiple comparison test. *p*-values less than 0.05 were considered statistically significant and were highlighted an asterisk (“*”). Abbreviations used include ACLD: advanced chronic liver disease; MASLD: metabolic dysfunction associated steatotic liver disease.

We analyzed the reasons for decompensation and the underlying causes of ACLD in relation to serum adiponectin levels. Sepsis was the precipitating event in 51.7% of cases, followed by gastrointestinal or other hemorrhage in 27.3% and non-septic bacterial infections in 11.2%. Upon ICU admission, patients with sepsis had significantly higher serum adiponectin levels compared to those with hemorrhage (*p* = 0.0045, Figure 2A). Notably, a subcohort analysis revealed significantly higher adiponectin concentrations in male patients with sepsis compared to those with hemorrhage (*p* = 0.0148), while no such difference was observed in female patients. Furthermore, patients were categorized based on the etiology of chronic liver disease, and serum adiponectin levels at ICU admission were analyzed. Patients with a viral etiology of ACLD and those with MASLD exhibited a trend towards higher serum adiponectin levels compared to other etiologies. However, this trend was not statistically significant (*p* = 0.1696, Figure 2B).

### 3.4. Serum Concentrations of Adiponectin Correlate with Markers of Hepatic Dysfunction

A comprehensive correlation analysis was performed to assess potential influences and covariates among clinical and laboratory parameters related to serum adiponectin concentrations. No significant correlation of adiponectin concentrations with BMI was found (Table 2). A moderate inverse correlation was identified between serum adiponectin and sodium concentrations (Spearman’s *r* = –0.2652, *p* = 0.001, Table 2). Furthermore, a moderately positive correlation was observed between total bilirubin and serum adiponectin concentrations (Spearman’s *r* = 0.29116, *p* < 0.001, Table 2). A mild negative correlation was noted between the fraction of inspired oxygen (FiO_2_) and serum adiponectin levels. Among the disease severity and clinical scores, the MELD score showed a moderately strong positive correlation with serum adiponectin levels (Spearman’s *r* = 0.27912, *p* < 0.001, Table 2). Similarly, a moderately strong positive correlation was detected between the Child–Pugh score points and serum adiponectin levels (Spearman’s *r* = 0.22967, *p* = 0.006, Table 2). Importantly, no correlation was found between serum adiponectin concentrations and scores used for the determination of organ failures, such as the Sequential Organ Failure Assessment (SOFA) score or the CLIF-C OF score.

**Figure 3:**
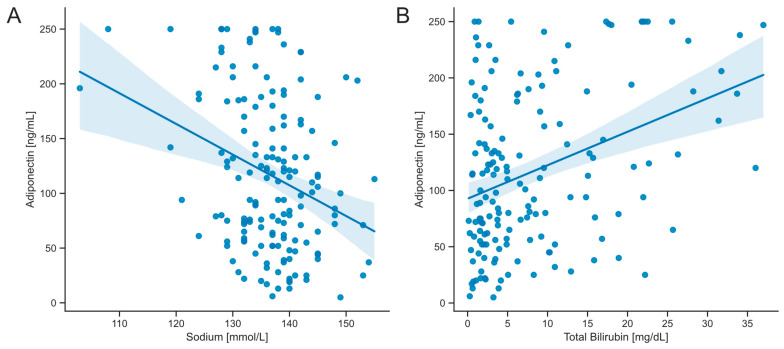
Correlation analysis of serum adiponectin levels. Scatter plots were created with a plotted linear regression fit between serum adiponectin levels and clinical parameters in all patients: (**A**) sodium and (**B**) total bilirubin. Regression analyses were conducted using univariate linear regression (see Table 3 for coefficients and *p*-values). The shaded areas in the plots represent the 95% confidence interval for the regression estimate.

To further investigate the relationship between correlated parameters and serum adiponectin levels, both univariate and multivariate linear regression analyses were conducted (Figure 3, Table 3). In these analyses, total bilirubin and sodium showed the most significant influence, with beta coefficients of 25.44 and −20.88, respectively (Table 3). In the univariate linear regression analysis, sodium, total bilirubin, MELD score, and Child–Pugh score points reached statistical significance and were subsequently included in the multivariate regression analysis (Table 3). Ultimately, only sodium and total bilirubin emerged as independent predictors of serum adiponectin concentrations (Figure 3, Table 3).

### 3.5. Serum Concentrations of Adiponectin Are Associated with Transplant-Free Survival in Critically Ill Patients with Acute Liver Dysfunction

Critically ill patients often experience disruptions in various physiological processes, which are linked to inflammatory responses and metabolic disorders. Adipocytokines, such as adiponectin, have been associated with disease severity and both short- and long-term mortality in critically ill patients [32]. This study aimed to elucidate the prognostic value of serum adiponectin concentration upon ICU admission in critically ill patients with liver disease.

**Figure 4:**
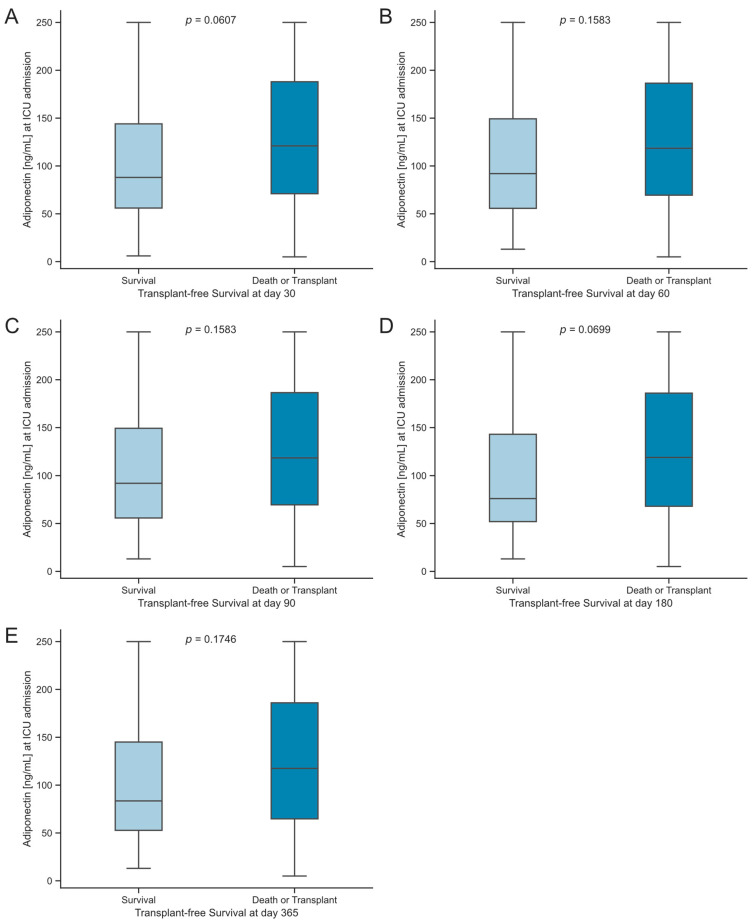
Consecutive survival analysis of critically ill patients. Adiponectin levels in a consecutive survival analysis of critically ill patients who were treated in the ICU. (**A**–**E**) Survival status from days 30 to 365. The sample size was patients *n* = 161. Significance between groups was evaluated using the Mann–Whitney U test. *p*-values < 0.05 were considered statistically significant.

Comparisons of serum adiponectin concentrations at ICU admission between surviving and those who either deceased or underwent transplantation at multiple time points (30, 60, 90, 180, and 365 days) did not show statistically significant differences (Figure 4). However, there was a consistent trend of slightly higher median serum adiponectin levels in deceased or transplanted patients (Figure 4A,D).

**Figure 5:**
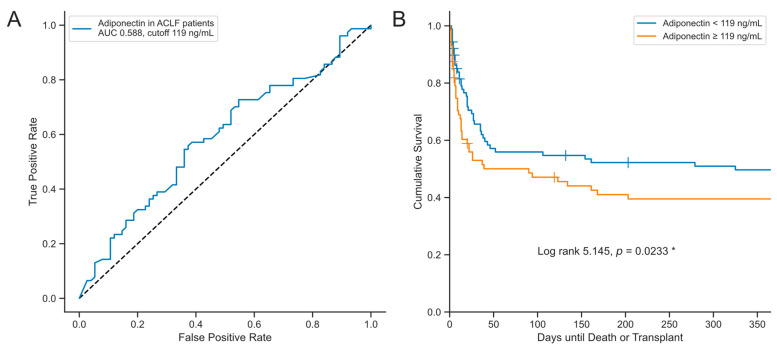
Analysis of transplant-free survival. (**A**) ROC curve for prediction of 30-day survival by serum adiponectin. Cutoff values were determined by the Youden index for ACLF patients. Sample sizes: patients *n* = 161. (**B**) The Kaplan–Meier curves depict survival probabilities based on serum adiponectin levels below 119 pg/mL (blue) and equal to or above 119 pg/mL (orange) for all patients. Censored events are indicated by vertical lines. The cutoff value for the Kaplan–Meier analysis was determined using the Youden index. Sample sizes: total patients *n* = 161, dACLD *n* = 21, ACLF *n* = 124, ALF *n* = 17. Statistical significance between groups was assessed using the log-rank test with *p*-values < 0.05 considered significant and highlighted with an asterisk (“*”). Abbreviations used include AUC: area under curve; ACLF: acute-on-chronic liver failure; ROC: receiver operating characteristic.

By utilizing Youden’s index, an optimal cut-off value for serum adiponectin concentration was determined at 119 ng/mL, balancing sensitivity and specificity (Figure 5A), although the predictive performance with an AUROC of 0.588 is low. Nevertheless, Kaplan–Meier analysis demonstrated that higher serum adiponectin levels at ICU admission were significantly associated with worse transplant-free survival (log-rank 5.145, *p* = 0.0233, Figure 5B). Next, univariate Cox regression analysis was performed to evaluate the association between dichotomized serum adiponectin levels at ICU admission and time until death or liver transplantation. Higher serum adiponectin levels at ICU admission were associated with decreased transplant-free survival in the entire cohort in a Cox regression analysis (HR: 1.002897; CI: 1.000179–1.005623, *p* = 0.037, Table 4). However, this association was not confirmed in the subgroup analysis of patients with ACLF or ALF (Table S3). Furthermore, the analysis suggested that this relationship was primarily driven by serum bilirubin concentrations (Table 4). The prognostic value was not enhanced by a combination of parameters (Table S4).

Male patients exhibited lower adiponectin serum concentrations upon ICU admission (Figure 1B). Consequently, a detailed analysis of sex-specific mortality prediction was performed. When comparing serum adiponectin levels at ICU admission between surviving and deceased or transplanted patients, no statistically significant differences were observed between males and females (Figure S2 and Table S2). To further investigate, Youden’s index was adjusted according to sex, revealing that the optimal cutoff for predicting mortality was lower in males (121 pg/mL) than in females (170 pg/mL). Additionally, Kaplan-Maier analysis indicated that higher adiponectin concentrations in females were associated with worse transplant-free survival (log-rank 5.794, *p* = 0.0161, Figure S3). However, the Cox regression analysis did not identify sex as a significant factor in mortality prediction (Table 4).

### 3.6. Stability of Serum Adiponectin Levels in the Early Stages of Critical Illness in Patients with Liver Disease

In addition, we investigated the clinical and prognostic significance of the serum adiponectin concentrations 48 h after ICU admission. Follow-up measurements were taken for 85 patients. The serum adiponectin levels after 48 h remained stable compared to the initial levels for patients with ACLF and ALF, while patients with dACLD showed a slightly higher median level. However, this difference was not statistically significant (Figure S4A). A paired analysis comparing serum adiponectin levels at ICU admission and after 48 h of treatment showed no significant changes within the patient cohorts (Figure S4B). To assess the potential impact of initial serum adiponectin regulation on survival, we compared levels at ICU admission and 48 h of treatment for surviving patients versus those who had passed away or received transplants by day 30. There were no statistically significant differences found (Figure S4C).

## 4. Discussion

Our study aimed to explore the correlation between serum adiponectin concentrations and liver dysfunction, investigating adiponectin as a potential biomarker in critically ill patients with decompensated advanced chronic liver disease, acute-on-chronic liver failure, and acute liver failure. We found that serum adiponectin levels were significantly elevated in patients with liver dysfunction compared to healthy controls. This aligns with the existing literature on adiponectin’s role in inflammation and metabolic processes associated with liver disease [1,2,28,33,34]. Previous studies have also shown elevated adipokine levels in patients with chronic liver disease [35,36,37], suggesting a possible influence of adiponectin levels in the development and progression of liver fibrosis. However, the mechanisms linking hyperadiponectinemia and liver fibrosis are not fully understood.

Elevated adiponectin levels have been reported in hepatitis C and non-alcoholic fatty liver disease [38]. We observed a tendency towards higher serum adiponectin levels in patients with viral cirrhosis compared to other etiologies. The literature shows contradictory findings: some studies report higher levels of chronic hepatitis C infections [39,40,41], while others find that chronic alcohol consumption leads to significantly higher adiponectin levels compared to viral hepatitis [42]. One study even links increasing adiponectin levels with alcohol consumption [43]. The discrepancy in our findings may be due to differences in the patient populations. Our cohort might include more patients with advanced liver disease or different stages of liver damage, which could affect adiponectin levels.

We observed a statistically significant correlation with bilirubin. This association aligns with findings from studies in non-critically ill patients and animal models, which suggest that adiponectin may be secreted through the biliary system [44,45]. Elevated bilirubin levels, often indicative of cholestasis or liver dysfunction, could be linked to increased adiponectin levels due to this potential biliary secretion.

Moreover, the study revealed a moderately strong negative correlation between serum adiponectin and sodium levels. These findings suggest that elevated adiponectin is associated with worsening liver function and hyponatremia, both critical factors in the prognosis of liver disease. Hyponatremia has been recognized as a predictor of poor outcomes in cirrhotic patients, which could explain its correlation with higher adiponectin levels [46,47]. Positive correlations between adiponectin levels and both MELD and Child–Pugh scores further support adiponectin as a marker of liver disease severity [44,48,49,50].

Importantly, there was no significant correlation between adiponectin levels and the Sequential Organ Failure Assessment (SOFA) score or the CLIF-C Organ Failure score. This suggests that adiponectin’s prognostic value may be more specific to liver-related parameters rather than overall organ failure. This hypothesis is supported by studies that have shown similar adiponectin levels in critically ill patients with or without sepsis compared to healthy controls [13,32]. Notably, patients with sepsis-induced decompensation of ACLD had significantly higher serum adiponectin levels than those with other precipitating events. This finding potentially indicates a complex interaction between liver dysfunction, systemic inflammation, and adiponectin regulation.

The univariate Cox regression analysis identified serum adiponectin levels at ICU admission as a significant prognostic factor for transplant-free survival in the overall cohort. However, this finding was not confirmed in the subgroups of patients with ACLF and ALF, likely due to the heterogeneous nature of liver diseases and varying mechanisms influencing adiponectin levels. Kaplan–Meier analysis supported the association of lower adiponectin levels with better transplant-free survival. A study of 40 patients with compensated advanced chronic liver disease found that serum adiponectin levels were not independently predictive of overall survival [51].

Interestingly, the optimal cutoff for predicting mortality was lower in males, and Kaplan–Maier analysis revealed the association of lower adiponectin values with better transplant-free survival in females but not in male patients with an adjusted cut-off. Despite this, the Cox regression did not identify sex as a significant prognostic factor. A sex dimorphism was reported for the association between adiponectin and cardiovascular mortality [52,53], suggesting a predictive value in males but not in females. In a recent study investigating adiponectin concentrations in 156 SIRS/septic patients, patients with cirrhosis as an underlying disease showed elevated adiponectin levels without differences in sex [28]. The study also revealed lower levels of adiponectin in female septic patients, which could be a possible explanation for the observed differences seen in the analysis regarding the precipitating event [28]. Moreover, higher adiponectin values in deceased male patients with sepsis were observed [28]. The predictive value of adiponectin may differ in disease entities. Further studies are necessary to validate adiponectin’s prognostic potential in advanced chronic liver disease and the here shown sex dimorphism.

This study has several limitations, including a relatively small sample size and single-center design, which may limit the generalizability of our findings. The observational nature of the study precludes establishing causality between adiponectin levels and clinical outcomes. Future research should aim to validate these findings in larger, multicenter cohorts and explore the mechanisms linking adiponectin to liver disease progression and outcomes. Additionally, investigating the interplay between adiponectin and other inflammatory and metabolic markers could provide further insights into the complex pathophysiology of liver dysfunction in critically ill patients.

In conclusion, our study demonstrates that serum adiponectin levels are elevated in critically ill patients with liver disease and are lined to transplant-free survival. The consistency of adiponectin levels during early ICU admission suggests its potential clinical value. Elevated serum adiponectin levels may indicate a higher risk of poorer transplant-free survival, underscoring the importance of additional research to confirm these results and investigate the mechanisms through which adiponectin affects the progression and outcomes of liver disease.

## Figures and Tables

**Table 1:** Baseline characteristics.

Parameter	dACLD	ACLF	ALF	*p*
Number, *n*	20	124	17	
Sex, female, *n* (%)	9 (45%)	50 (40.3%)	9 (52.9%)	0.5924
Age (years)	59.5 (15.25)	58 (15.25)	52 (32)	0.1947
BMI (kg/m^2^)	23.1 (4.5)	27.6 (8.5)	25.7 (6.5)	0.0044 *
APACHE II score	16.5 (12)	27 (14)	15 (11)	<0.001 *
SOFA score	8 (4)	14 (5)	9 (3)	<0.001 *
MELD score	13 (6)	28 (12.25)	30 (11)	<0.001 *
Liver transplantation, *n* (%)	3 (15.0%)	13 (10.5%)	6 (35.5%)	0.0199 *
Mechanical ventilation, *n* (%)	3 (15.0%)	64 (51.6%)	2 (11.8%)	<0.001 *
Vasopressor demand, *n* (%)	1 (5.0%)	100 (80.6%)	4 (23.5%)	<0.001 *
ICU days, *n*	3 (1.25)	6 (10)	6 (11)	<0.001 *
Death in ICU, *n* (%)	0 (0%)	70 (56.5%)	4 (23.5%)	<0.001 *
30-day mortality, *n* (%)	0 (0%)	60 (52.2%)	2 (11.8%)	<0.001 *
1-year mortality, *n* (%)	3 (15.8%)	76 (66.7%)	5 (31.2%)	<0.001 *
Adiponectin (ng/mL)	67.5 (101)	113.5 (117.5)	117 (87)	0.2974

Data are presented as median values with IQR in parentheses unless specified otherwise. Statistical significance among dACLD, ACLF, and ALF patient groups was determined using the Kruskal–Wallis test or chi-squared test as appropriate. A *p*-value of less than 0.05 was considered statistically significant, highlighted with an asterisk (“*”). Abbreviations used include dACLD: decompensated advanced chronic liver disease; ACLF: acute-on-chronic liver failure; ALF: acute liver failure; IQR: interquartile range; APACHE: Acute Physiology and Chronic Health Evaluation; SOFA: Sequential Organ Failure Assessment; ICU: intensive care unit.

**Table 2:** Correlations of clinical and laboratory parameters with adiponectin serum concentrations at ICU admission.

Parameter	Spearman’s *r*	*p*
Demographics		
Age	−0.0072	0.928
BMI	0.11449	0.149
Blood count and markers of inflammation		
WBC	−0.059	0.457
Hemoglobin	0.09237	0.244
Platelets	0.00758	0.924
CRP	−0.00721	0.364
PCT	0.03524	0.672
IL-6	0.02512	0.781
Electrolytes and renal system		
Sodium	−0.2652	0.001 *
Potassium	−0.0659	0.407
pH	0.12674	0.109
Urea	−0.0002	0.998
Creatinine	0.10018	0.206
eGFR	−0.1247	0.115
Diuresis per day	0.0925	0.252
Coagulation and hepato-pancreatico-biliary system		
Albumin	−0.1243	0.116
INR	0.15482	0.05
Bilirubin, total	0.29116	<0.001 *
AST	0.03012	0.705
ALT	0.08779	0.268
gGT	−0.1174	0.138
AP	0.14077	0.075
Cholesterol	−0.0958	0.237
Cardiopulmonary system		
NT-pro BNP	−0.0891	0.31
Norepinephrine demand	−0.0931	0.345
Horovitz quotient (P_a_O_2_/F_i_O_2_)	0.14989	0.058
F_i_O_2_	−0.1844	0.019 *
Lactate	0.09991	0.207
Disease severity and clinical scores		
Length of stay in ICU	−0.0246	0.757
Length of stay in hospital	0.04102	0.605
SOFA score	0.05352	0.5
APACHE II score	−0.0302	0.703
SAPS II score	−0.0817	0.303
MELD score	0.27912	<0.001 *
Child–Pugh points	0.22967	0.006 *
CLIF-C OF score	0.0506	0.556
CLIF-C ACLF score	0.06008	0.507

The Spearman rank correlation test was employed to assess both positive and negative correlations, with statistical significance defined as *p*-values < 0.05 (highlighted with an asterisk (“*”). Abbreviations used include BMI: body mass index; WBC: white blood cell count; CRP: C-reactive protein; PCT: Procalcitonin; IL-6: Interleukin 6; GFR: Glomerular filtration rate; INR: international normalized ratio; AST: Aspartate aminotransferase; ALT: Alanine aminotransferase; gGT: gamma-glutamyltransferase; AP: alkaline phosphatase; BNP: N-terminal pro-B-type natriuretic peptide; FiO2: Fraction of inspired oxygen; ICU: intensive care unit; SOFA: Sequential organ failure assessment; APACHE II: acute physiology and chronic health evaluation II; MELD: Model of End-Stage Liver Disease; CLIF-C OF: Chronic Liver Failure Consortium (CLIF-C) organ failure score; CLIF-C ACLF: Chronic Liver Failure Consortium (CLIF-C) ACLF score.

**Table 3:** Linear univariate and multivariate regression.

	Univariate Regression				Multivariate Regression		
Covariate	β Coefficient	Coefficient	95 % CI	*p*	Coefficient	95 % CI	*p*
Sodium	−20.88	−2.8030	−4.2150–−1.3910	<0.001 *	−2.6195	−4.007–−1.232	<0.001 *
Bilirubin, total	25.44	2.9733	1.7727–4.1739	<0.001 *	1.7467	0.226–3.267	0.025 *
MELD score	18.92	2.1481	0.9433–3.3528	<0.001 *	0.8322	−0.734–2.398	0.295
F_i_O_2_	−10.26	−0.4424	−0.9125–0.0276	0.0649			
Child–Pugh points	17.57	8.4181	2.9556–13.8812	0.003 *	2.3704	−3.813–8.554	0.450

Uni- and multivariable linear regression analyses were performed to evaluate serum adiponectin concentrations across all patients, incorporating demographic factors and relevant laboratory parameters such as sodium, total bilirubin, MELD Score, FiO_2_, and Child–Pugh Score. Variables that reached statistical significance in the univariate analysis were included in the multivariate regression. Standardized beta coefficients were calculated to ensure comparability between variables. Significance was determined using a linear regression model with *p*-values < 0.05 considered statistically significant and highlighted with an asterisk (“*”). Abbreviations used include MELD: Model of End-Stage Liver Disease; FiO_2_: fraction of inspired oxygen.

**Table 4:** Cox regression for transplant-free survival for all patients.

Covariate	Hazard Ratio	95 % CI	*p*
Adiponectin	1.002897	1.00179–1.005623	0.0367 *
Age	1.001946	0.988774–1.015295	0.7734
Sex	0.873853	0.589472–1.1595429	0.5020
Sex, male	1.002670	0.998934–1.006420	0.1612
Sex, female	1.003799	0.999622–1.007995	0.0747
Sodium	1.010828	0.984609–1.037745	0.4219
Bilirubin	1.053230	1.031838–1.075065	<0.001*
APACHE II score	1.028431	1.009198–1.048031	0.0036 *

Univariate Cox regression analysis was conducted. Significance was determined using a linear regression model. *p*-values less than 0.05 were considered statistically significant and were denoted with an asterisk (“*”). Abbreviations used include CI: confidence interval.

## Data Availability

The data supporting the findings of this study are available from the corresponding author upon reasonable request. However, certain data may not be accessible due to privacy or ethical concerns.

## Supplementary Materials

The following supporting information can be downloaded at: https://www.mdpi.com/article/10.3390/biomedicines12102173/s1, Figure S1: Comparison of serum adiponectin levels in ACLF grades; Figure S2: Consecutive survival analysis for transplant free survival with regard to sex; Figure S3: Sex dependent Kaplan Maier analysis; Figure S4: Analysis of serum adiponectin levels 48 h after admission to ICU; Table S1: Comorbidities and their influence on adiponectin levels; Table S2: Consecutive survival analysis for transplant free survival with regard to sex; Table S3: Cox regression for transplant-free survival; Table S4: multivariate Cox-regression model.
