# Supplementary material for: Serum Adiponectin Is Elevated in Critically Ill Patients with Liver Disease and Associated with Decreased Overall Survival
Source: Biomedicines. 2024 Sep 25;12(10):2173. doi: 10.3390/biomedicines12102173 (PMC11504267; doi:10.3390/biomedicines12102173)
Supplement: Supplementary file 1 [file biomedicines-12-02173-s001.zip › biomedicines-3220124-supplementary.pdf]

Supplementary

Serum Adiponectin Is Elevated in Critically Ill Patients with Liver Disease and Associated with Decreased Overall Survival

Maike R. Pollmanns [1], Qendrim Pajaziti [1], Philipp Hohlstein [1], Jule K. Adams [1], Samira Abu Jhaisha [1], Elena Kabak [1], Karim Hamesch [1], Sophie H. A. Nusser [1], Ralf Weiskirchen [2], Theresa H. Wirtz [1,†] and Alexander Koch [1,*,†]

Table of contents:

Figure S1: Comparison of serum adiponectin levels in ACLF grades

Figure S2: Consecutive survival analysis for transplant free survival with regard to sex

Figure S3: Sex dependent Kaplan Maier analysis

Figure S4: Analysis of serum adiponectin levels 48 hours after admission to ICU

Table S1: Comorbidities and their influence on adiponectin levels

Table S2: Consecutive survival analysis for transplant free survival with regard to sex

Table S3: Cox-Regression for transplant-free survival

Table S4: multivariate Cox-regression model

Figure S1: Comparison of serum adiponectin levels in ACLF grades

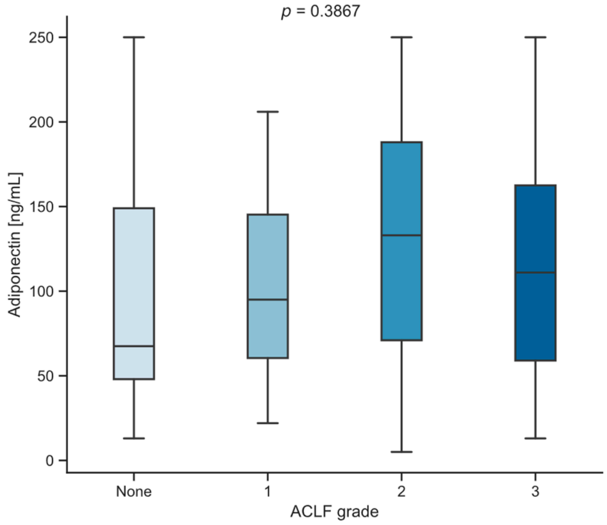

Serum adiponectin levels were compared among all study subjects across different ACLF grades. * Significance between groups was evaluated using the Kruskal-Wallis test. p-values < 0.05 were considered statistically significant. *Abbreviations are:* ACLF: Acute-on-Chronic Liver Failure.

Figure S2: Consecutive survival analysis for transplant free survival with regard to sex

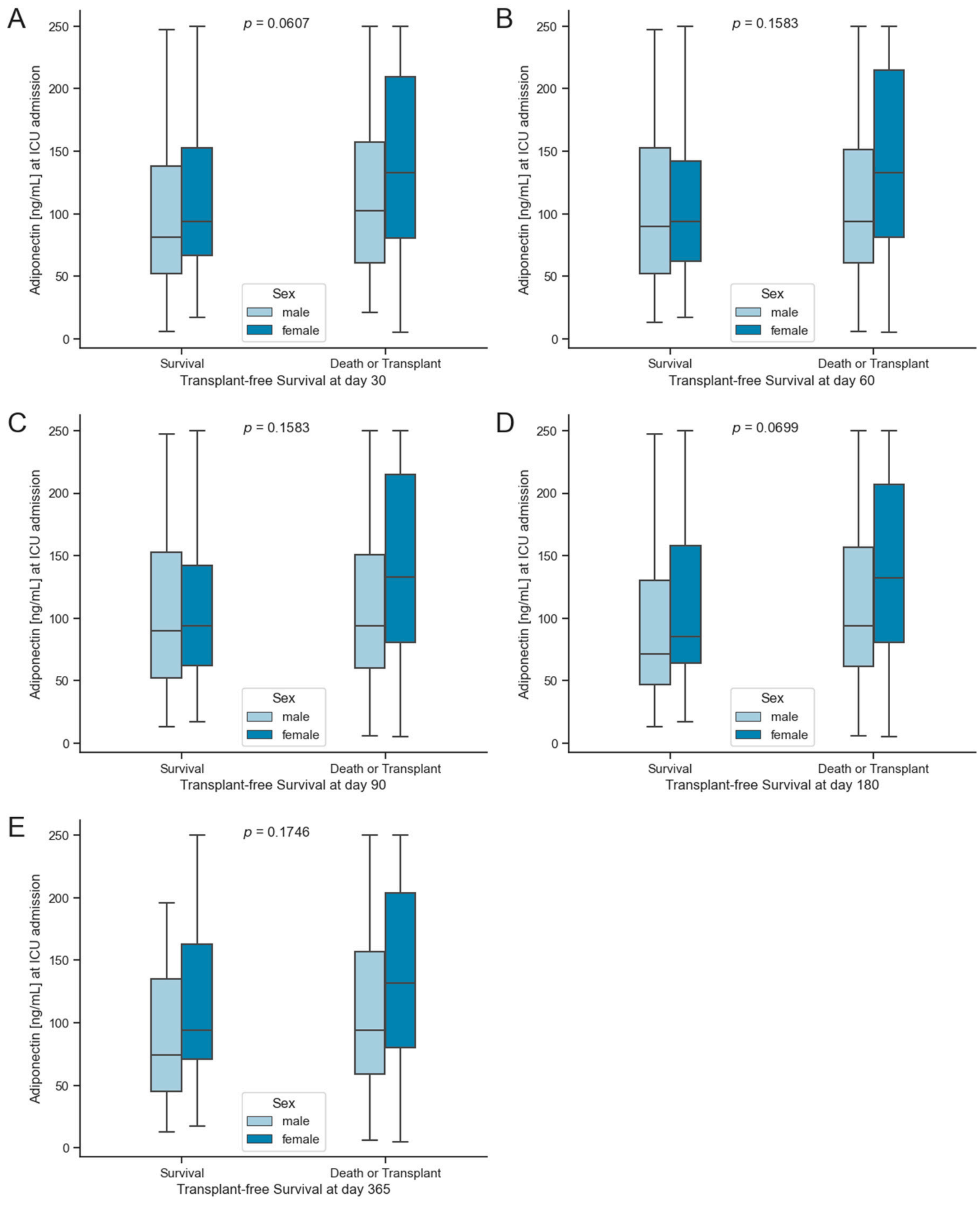

Adiponectin levels in a consecutive survival analysis of critically ill patients who were treated in the ICU with subgroup analysis regarding the sex. (A-E) Survival status from days 30 to 365. The sample size was: patients n = 161. Significance between groups was evaluated using the Mann-Whitney U test. p-values < 0.05 were considered statistically significant.

Figure S3: Sex dependent Kaplan Maier analysis

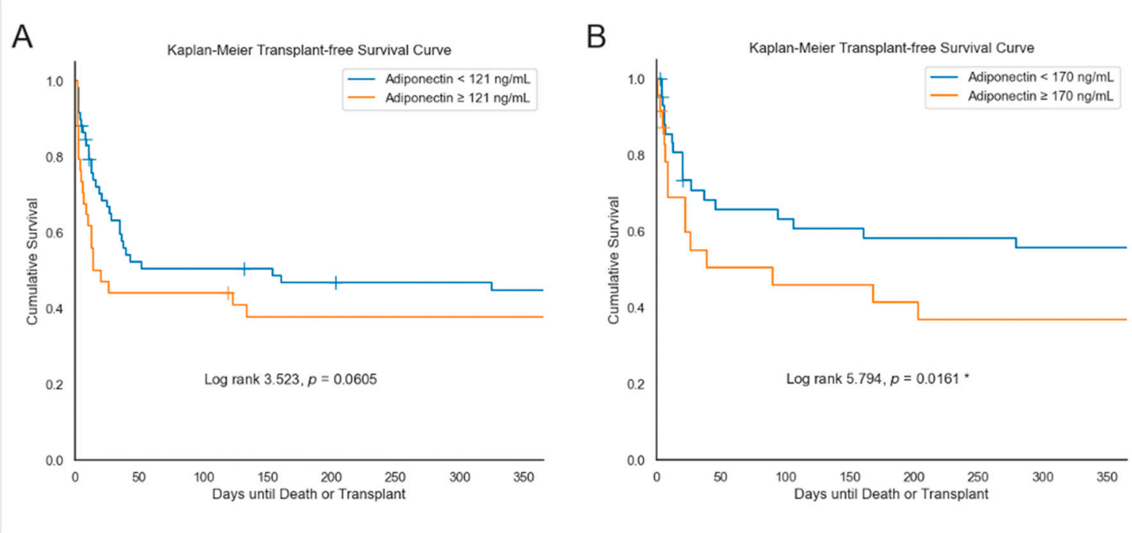

The Kaplan-Meier curves depict survival probabilities based on serum adiponectin levels. (A) Subgroup analysis of male patients. Optimal cutoff was determined by Youden's index: below 121 pg/mL (blue) and equal to or above 121 pg/mL (orange) for all male patients. Sample size: $n = 93$. (B) Subgroup analysis of female patients. Optimal cutoff was determined by Youden's index: below 170 pg/mL (blue) and equal to or above 170 pg/mL (orange) for all female patients. Sample size: $n = 68$. Censored events are indicated by vertical lines. Statistical significance between groups was assessed using the log rank test with p-values < 0.05 considered significant.

Figure S4: Analysis of serum Adiponectin levels 48 hours after admission to ICU

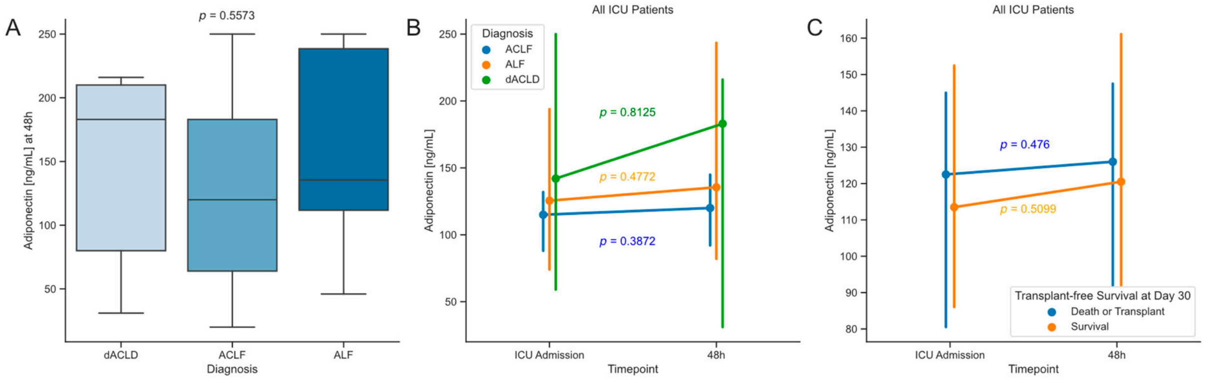

(A) Adiponectin levels 48 hours after admission to the ICU in critically ill patients with liver dysfunction. **(B)** Analysis of the dynamics of serum adiponectin levels between the day of admission to ICU and 48 hours after admission in relation to the diagnosis. **(C)** Comparison of serum adiponectin concentrations at ICU admission to levels after 48 hours of treatment between surviving and deceased or transplanted patients at day 30. Vertical error bars indicate the 95% confidence intervals around the median **(B,C)**. Sample sizes: patients n = 85. Significance between groups was assessed using the Kruskal-Wallis test for more than two groups or the Wilcoxon signed rank test for paired samples. *p*-values < 0.05 were considered statistically significant. *Abbreviations are*: ICU: intensive care unit; dACLD: decompensated advanced chronic liver disease; ACLF: acute-on-chronic liver failure; ALF: acute liver failure.

Table S1: Comorbidities and their influence on adiponectin levels

**Comorbidity**	**Adiponectin concentration in ng/mL, median (range)**	***p***
**Diabetes mellitus (n = 42)**	121.0 (6.0 - 250.01)	0.8172
**Hypertension (n = 47)**	84.5 (6.0 - 250.01)	0.067
**Coronary artery disease (n = 19)**	83.5 (6.0 - 229.0)	0.1462
**Chronic alcohol consumption (n = 86)**	108.0 (6.0 - 250.01)	0.3426
**Chronic obstructive pulmonary disease (n = 12)**	94.0 (37.0 - 236.0)	0.7281

**Malignancy (n = 21)**	100.0 (19.0 - 250.01)	0.5469
**Hepatocellular carcinoma (n = 8)**	129.0 (40.0 - 250.01)	0.7001
**Hematological neoplasm (n = 6)**	128.5 (19.0 - 194.0)	0.9608
**Solid neoplasm (n = 7)**	94.0 (20.0 - 180.0)	0.3706

The median and range (in parentheses) are given, unless otherwise indicated. *Significance between groups was assessed using the Mann-Whitney U test. *p*-values < 0.05 were considered statistically significant and were marked with an asterisk ("*").

Table S2: Consecutive survival analysis for transplant free survival with regard to sex

	**male**	**females**
**Day 30**	0.1965	0.2
**Day 60**	0.4842	0.15
**Day 90**	0.4842	0.15
**Day 180**	0.1622	0.1916
**Day 365**	0.2219	0.3434

Comparison of the sex in survival analysis for transplant free survival in critically ill patients who were treated in the ICU. Significance between groups was assessed using the Mann-Whitney U test. *p*-values < 0.05 were considered statistically significant and were marked with an asterisk ("*").

Table S3: Cox-Regression for transplant-free survival

	**Hazard ratio**	**Lower 95%**	**Upper 95%**	***p***
**All patients**	1.002897	1.000179	1.005623	0.037 *
**ACLF patients**	1.001601	0.998584	1.004628	0.297
**ALF patients**	1.010406	0.999077	1.021863	0.072

Univariate Cox-Regression analysis. Significance was assessed using a linear regression model. p-values < 0.05 were considered statistically significant and were

highlighted ("*"). *Abbreviations are*: ACLF: acute-on-chronic liver failure; ALF: acute liver failure.

Table S4: multivariate Cox-regression model

	**Hazard ratio**	**Lower 95 %**	**Upper 95 %**	***p***
**Adiponectin**	1.002967	0.999962	1.005439	0.0533
**APACHE II score**	1.027321	1.008161	1.046845	0.005 *

Multivariate Cox-Regression model. Significance was assessed using a linear regression model. p-values < 0.05 were considered statistically significant and were highlighted ("*"). *Abbreviations are*: APACHE II: acute physiology and chronic health evaluation II
